# Serum lactate dehydrogenase activities as systems biomarkers for 48 types of human diseases

**DOI:** 10.1038/s41598-021-92430-6

**Published:** 2021-06-21

**Authors:** Yuling Wu, Caixia Lu, Nana Pan, Meng Zhang, Yi An, Mengyuan Xu, Lijuan Zhang, Yachong Guo, Lijuan Tan

**Affiliations:** 1 Systems Biology and Medicine Center for Complex Diseases, Center for Clinical Research, Affiliated Hospital of Qingdao University, Qingdao, 266003 China; 2 Department of Cardiology, The Affiliated Hospital of Qingdao University, Qingdao, 266003 China; 3 Kuang Yaming Honors School, Nanjing University, Nanjing, 210023 China; 4 Institute Theory of Polymers, Leibniz-Institut Für Polymerforschung Dresden, 01069 Dresden, Germany

**Keywords:** Diagnostic markers, Biomarkers

## Abstract

Most human diseases are systems diseases, and systems biomarkers are better fitted for diagnostic, prognostic, and treatment monitoring purposes. To search for systems biomarker candidates, lactate dehydrogenase (LDH), a housekeeping protein expressed in all living cells, was investigated. To this end, we analyzed the serum LDH activities from 172,933 patients with 48 clinically defined diseases and 9528 healthy individuals. Based on the median values, we found that 46 out of 48 diseases, leading by acute myocardial infarction, had significantly increased (*p* < 0.001), whereas gout and cerebral ischemia had significantly decreased (*p* < 0.001) serum LDH activities compared to the healthy control. Remarkably, hepatic encephalopathy and lung fibrosis had the highest AUCs (0.89, 0.80), sensitivities (0.73, 0.56), and specificities (0.90, 0.91) among 48 human diseases. Statistical analysis revealed that over-downregulation of serum LDH activities was associated with blood-related cancers and diseases. LDH activities were potential systems biomarker candidates (AUCs > 0.8) for hepatic encephalopathy and lung fibrosis.

## Introduction

Most of the aging-associated human diseases are systems diseases caused by multiple genetic and environmental factors in addition to life styles^1^. Patients usually take several prescription medicines to deal with various problems associated with systems diseases. To determine the overall effect of the medications, a blood-based biomarker that reflects the improvement of whole-body systems is preferred. We named such a biomarker a “systems biomarker”^2^.

The OMICS-based approaches, not limited to genomics, epigenomics, proteomics, glycomics, lipidomics, and metabolomics, have been used to developing a biomarker panel to define systems diseases^3,4^. However, none of the biomarker panel has been introduced into clinical practice during the past 30 years. Thus, we hypothesized that the biomarkers with the systems' characteristics might be found in the current clinical blood tests. Therefore, we have taken the “Lab Resultomics” approach for systems biomarker discovery^5,6^.

By analyzing the mean plus median levels, *p* values, and dynamic ranges of 17 clinical blood tests including 10 cancer biomarkers SCCA, CA724, CA50, CA242, CA125, CA199, CA153, AFP, CEA, and PSA^7^ from 1.4 million clinical samples in 64 human diseases, our published data showed that most of abnormal lab results including increased serum cancer biomarker levels are indicators of systems malfunction unrelated to cancer cells^2,5^. For example, increased serum SCCA levels are clinically used diagnostic or prognostic biomarkers for squamous cell carcinomas, we found that patients suffering uremia, azotemia, diabetic nephropathy, and nephritic syndrome have the highest serum SCCA levels among 39 different types of diseases, including patients suffering squamous cell carcinomas. Thus, SCCA is not a cancer cell-specific product, and failed clearance of serum SCCA explains the high SCCA levels in different types of human kidney diseases^8^. Thus, the “Lab Resultomics” approach represents a quick way to discover novel systems biomarker candidates for human diseases.

LDH is a housekeeping protein expressed in all living cells, with the highest activities found in the heart, liver, muscles, kidneys, lungs, and blood cells. LDH plays an essential role in glycolysis and gluconeogenesis by catalyzing the reversible conversion of lactate to pyruvate with concomitant interconversion of NADH and NAD^+^ as an oxidoreductase^9^. LDH consists of tetramers formed by two types of subunits: muscle (M) and heart (H). Based on the component of the subunits, LDH is divided into five isomeric types from LDH-1 to LDH-5 with variable enzymatic activities measured by in vitro assay. However, the serum LDH activities and their dynamic ranges have never been systematically studied and compared in different types of human diseases.

In the current study, the data of serum LDH activities from 172,933 patients with 48 clinically defined diseases and 9528 healthy individuals who attend their annual physical examination over the past 5 years were retrieved from the clinical laboratory of the Affiliated Hospital of Qingdao University. Different statistical methods were used for data analysis.

## Results

Based on the data retrieved, we calculated and listed the number of cases, median (interquartile ranges), mean (standard deviation, SD) and *p* value in comparison to healthy controls for each of the 48 diseases in Table 1. “*p* value” is an expression that is related to the significant difference between groups.

**Table 1:** Serum LDH activities (U/L) in 48 different types of clinical defined human diseases and healthy controls.

LDH	# of cases	Mean (SD)	Median (IQR)	*p* Value
Acute myocardial infarction	2639	331.9 (208.5)	255.0 (184.0, 411.5)	< 0.001
Hepatic encephalopathy	91	252.8 (93.8)	232.0 (191.0, 297.5)	< 0.001
Preeclampsia	992	237.0 (88.2)	216.0 (174.0, 276.0)	< 0.001
Lung fibrosis	336	236.3 (93.3)	209.0 (172.0, 270.0)	< 0.001
Liver cancer	299	219.7 (67.9)	206.0 (167.0, 257.0)	< 0.001
Myeloproliferative disorder	1294	243.2 (134.1)	206.0 (163.0, 273.8)	< 0.001
Lupus erythematosus	1333	216.4 (65.1)	201.0 (168.0, 249.0)	< 0.001
Nephrotic syndrome	3822	214.7 (64.4)	201.0 (165.0, 248.8)	< 0.001
Aplastic anemia	887	203.9 (60.0)	195.0 (164.0, 239.5)	< 0.001
Rheumatic arthritis	486	200.6 (51.8)	192.5 (162.0, 227.8)	< 0.001
Diabetic nephropathy	605	200.7 (48.8)	192.0 (163.0, 233.0)	< 0.001
Brain trauma	659	223.8 (120.9)	192.0 (142.0, 255.0)	< 0.001
Anemia	2063	221.4 (119.5)	190.0 (152.5, 245.0)	< 0.001
Uremia	6547	195.7 (52.0)	186.0 (158.0, 223.0)	< 0.001
Lymphoma	4811	209.8 (86.6)	185.0 (155.0, 234.0)	< 0.001
Cirrhosis	9328	198.7 (64.1)	185.0 (153.0, 230.0)	< 0.001
Sepsis	109	232.0 (121.8)	184.0 (144.0, 290.0)	< 0.001
Psoriasis	146	188.0 (45.3)	183.0 (155.0, 214.8)	< 0.001
Azotemia	497	192.7 (55.0)	180.0 (151.0, 221.0)	< 0.001
Lung cancer	10,943	188.7 (51.9)	178.0 (152.0, 212.0)	< 0.001
Leukemia	5416	224.3 (140.9)	176.0 (140.0, 247.0)	< 0.001
Breast cancer	5509	181.0 (36.4)	175.0 (154.0, 202.0)	< 0.001
Ovarian cancer	2357	184.3 (45.4)	175.0 (153.0, 205.0)	< 0.001
Intracranial hemorrhage	3839	192.6 (69.8)	173.0 (142.0, 225.0)	< 0.001
Hepatitis	6832	178.8 (41.1)	171.0 (148.0, 200.0)	< 0.001
Chronic obstructive PD	1651	186.6 (59.1)	171.0 (145.0, 210.0)	< 0.001
Bone fracture	1783	184.3 (54.9)	171.0 (144.0, 209.0)	< 0.001
Encephalitis	554	188.4 (70.4)	171.0 (141.0, 216.0)	< 0.001
Endometrial cancer	1217	175.7 (36.5)	170.0 (149.0, 194.0)	< 0.001
Kidney cancer	1553	180.9 (47.3)	170.0 (147.0, 204.0)	< 0.001
Pancreatitis	1835	209.5 (109.3)	170.0 (138.0, 240.0)	< 0.001
Nephritis	2156	180.7 (52.0)	168.0 (142.0, 205.0)	< 0.001
Healthy controls > 65 years old	811	167.8 (25.4)	167.0 (149.5, 187.0)	–
Coronary heart disease	22,077	182.3 (59.8)	167.0 (142.0, 204.0)	< 0.001
Multiple myeloma	2330	181.4 (65.4)	167.0 (139.0, 204.0)	< 0.001
Ankylosing spondylitis	103	185.0 (60.2)	165.0 (143.0, 218.0)	< 0.001
Cerebral arteriosclerosis	821	171.9 (39.2)	164.0 (144.0, 192.0)	< 0.001
Gastritis	3401	169.8 (35.4)	164.0 (144.0, 189.0)	< 0.001
Asthma	602	178.6 (56.3)	163.0 (143.0, 195.8)	< 0.001
Colon cancer	6644	173.6 (49.9)	163.0 (139.0, 197.0)	< 0.001
Pancreatic cancer	1150	173.9 (50.1)	163.0 (138.0, 200.0)	< 0.001
Gastric cancer	13,497	167.7 (40.0)	161.0 (139.0, 189.0)	< 0.001
Cervical cancer	2276	161.9 (31.1)	158.0 (139.0, 180.3)	< 0.001
Type 2 diabetes mellitus	11,548	165.4 (38.4)	158.0 (137.0, 186.0)	< 0.001
Rectum cancer	8498	166.7 (42.3)	158.0 (136.0, 188.0)	< 0.001
Esophagus cancer	4025	165.6 (42.5)	157.0 (135.0, 187.0)	< 0.001
Acute cerebral infarction	9624	166.9 (47.3)	156.0 (134.0, 186.0)	< 0.001
**Healthy controls**	**9528**	**156.2 (25.3)**	**153.0 (137.0, 173.0)**	**–**
Cerebral ischemia	2276	155.6 (32.7)	150.0 (132.0, 173.0)	< 0.001
Gout	1472	149.3 (32.3)	144.0 (125.0, 168.0)	< 0.001

The healthy control is bolded for easy comparison to the different types of diseases listed.

*SD* standard deviation, *IQR* interquartile range, *Chronic Obstructive PD* Chronic Obstructive Pulmonary Disease.

**Figure 1:**
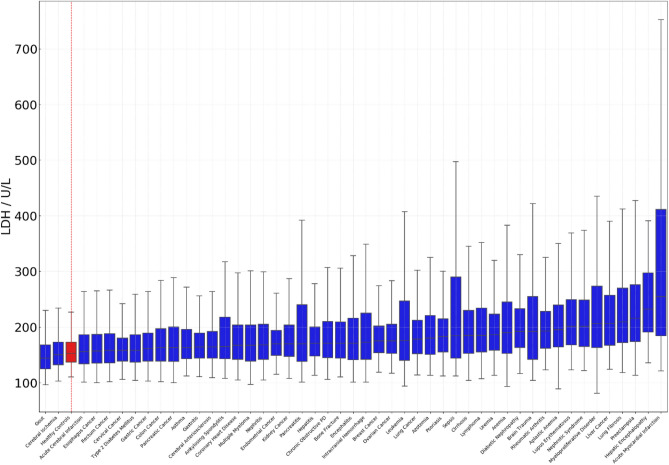
Serum LDH activities in 48 different types of diseases. The average in red would be that of the control group. The data were sorted in ascending order according to the median values. *Chronic obstructive PD* chronic obstructive pulmonary disease.

To visualize the results, the boxed plots of serum LDH activities with lower quartile (25%), median (50%), upper quartile (75%) ranges, and 95% confident intervals marked for each of the 48 diseases in addition to the healthy control were drawn and shown in Fig. 1. The patients suffering gout and cerebral ischemia had lower, while the other 46 types of diseases had higher serum LDH activities than the healthy control with statistical significance (*p* < 0.001, Table 1). Moreover, among the 48 diseases studied, patients with acute myocardial infarction had the highest median level of serum LDH activities.

Interestingly, blood-related cancers and diseases, including myeloproliferative disorders, aplastic anemia, anemia, leukemia, and multiple myeloma, were accompanied by significantly higher and lower LDH activities than the healthy control and other diseases. The significant high and low LDH activities were the characteristics of specific diseases. Such LDH activities were not observed in the diseases with larger numbers of tested cases (Table 1), such as lung cancers, coronary heart disease, type 2 diabetes, or diseases with comparable numbers of tested cases to that of blood-related cancers and diseases.

**Figure 2:**
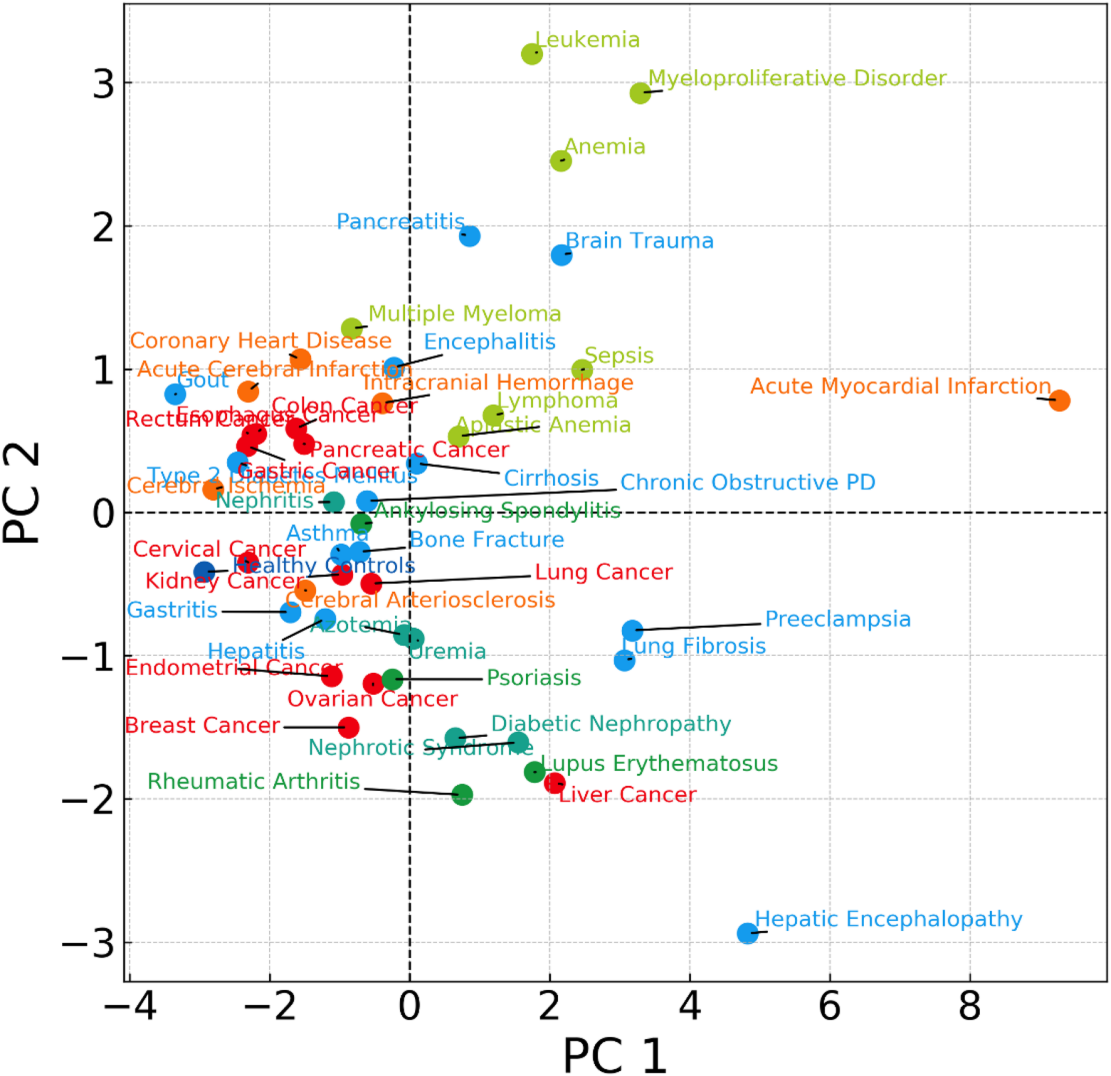
The changes in serum LDH activitie had common features for the same class of diseases. PC means Principal Component. The 48 diseases were divided into six major classes, including solid cancers (marked in red), autoimmune diseases (marked in green), cardiovascular and cerebrovascular diseases (marked in orange), acute diseases (marked in blue), blood-related diseases (marked in yellowish-green), and kidney diseases (bluish-green). The statistics features of the LDH activities for each of 48 diseases, including the mean, standard deviation, min/max value, 25, 50, and 75 percentiles were quantified. The obtained statistics features were further decoupled into two major components and presented.

To understand the heterogeneity of LDH activities among different diseases, we first divided 48 diseases into six major classes: solid cancers, autoimmune diseases, cardio- and cerebrovascular diseases, blood-related cancers and diseases, kidney diseases, and others. We then quantified the statistics features of the LDH activities for each of 48 diseases, including the mean, standard deviation, min/max value, 25, 50, and 75 percentiles. The obtained statistics features of all diseases were further decoupled into two major components presented in Fig. 2.

Apparent clustering of the same class of human diseases, such as solid cancers (at the left side of chart), autoimmune disease (at the low left side of the chart), blood-related cancers, and diseases (at the upper right side of the chart), were observed based on the statistical analysis. Except for acute myocardial infarction, most cardio- and cerebrovascular diseases were also clustered at the upper left side of the chart. The two major component analyses indeed clustered the blood-related cancers and diseases with up- and down-regulating blood LDH activities together. Interestingly, brain trauma and acute myocardial infarction associated with blood clotting were also located in the same area of the blood-related cancers and diseases (upper right corner), even though no extreme low LDH activities were present in the two diseases.

**Figure 3:**
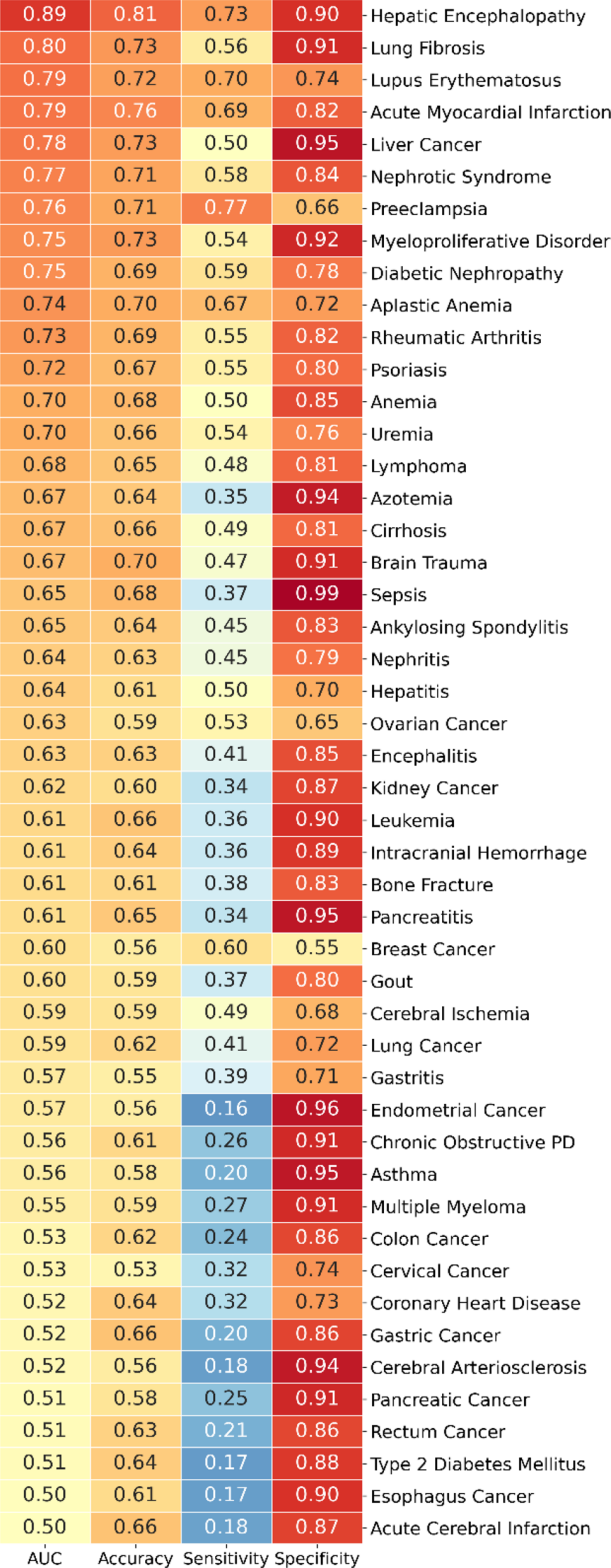
The AUC, accuracy, sensitivity, and specificity of serum LDH activities for 48 types of human diseases. *Chronic obstructive PD* chronic obstructive pulmonary disease, *AUC* area under the curve.

Lastly, we investigated the diagnostic properties of serum LDH activities as system biomarkers. We performed the receiving operator curve (ROC) analysis for all 48 types of diseases (Supplemental Fig. S1). The area under the curve (AUC), accuracy, sensitivity (Sen), and specificity (Spe) were summarized in Fig. 3 according to the descending orders of the AUC values of the diseases.

Among the 48 types of diseases tested, 31 of them had their AUCs over 0.60 (ranged from 0.60 to 0.89). Unexpectedly, the LDH activity served as the best biomarker for hepatic encephalopathy with an AUC of 0.89, sensitivity 73%, and specificity 90% among 48 human diseases, which were followed by lung fibrosis (AUC = 0.80), lupus erythromatus (AUC = 0.79), and acute myocardial infarction (AUC = 0.79). In contrast, the LDH activities had the lowest AUCs for esophagus cancer and acute cerebral infarction.

## Discussion and conclusion

Forty-six out of 48 diseases tested in the current study had increased serum LDH activities (Table 1), indicating LDH was a common disease biomarker. LDH concentrations in various tissues are higher than average physiological serum concentrations, approximately 5000–15,000 times^10^, so it is easy to see large deviations among various diseases even with minimal tissue damage. Most of the published reports investigated the biomarker properties of serum LDH activity in one specific disease using serum LDH activities from healthy individuals as control^11–22^, our work reported the first systematical comparison of serum LDH activities as biomarkers in 48 types of diseases.

Remarkably, gout and cerebral ischemia were the only two diseases whose median LDH activities were significantly lower than the healthy control. Gout is progressed by forming monosodium urate deposits in bone joints, kidneys, and subcutaneous sites. The monosodium urate crystals cause acute/chronic inflammation and tissue injury, eventually leading to chronic arthropathy^24^. Markus et al. suggested that synovial LDH activities could be an excellent diagnostic candidate to differentiate septic and gouty arthritis^25^. They proposed that the vascular leakages of serum uric acid and LDH to bone joints might be associated with gouty arthritis. This mechanism explained the low serum LDH activities in gout (Table 1 and Fig. 1). Interestingly, the AUC, accuracy, sensitivity, and specificity for gout were 0.60, 0.59, 0.37, and 0.80, respectively (Fig. 3), indicating that the serum LDH activity could serve as a decent systems biomarker candidate for gout when combined with other lab results.

Serum LDH was among the first diagnostic biomarker established for myocardial infarction^26^. Subsequently, LDH was also reported as a biomarker for different diseases. Even though LDH is not as effective as cardiac troponin in diagnosing acute myocardial infarction, elevated levels of LDH can be helpful in determining whether a patient has had a myocardial infarction if they come to doctors several days after an episode of chest pain.

Statistically, *p* value is very low when higher numbers of samples are analyzed. Indeed, all *p* values were less than 0.001 for all 48 diseases compared to the healthy control, as shown in Table 1. In contrast, sensitivity and specificity are significantly lowered with a higher number of samples analyzed for the cancer biomarkers^23^. Thus, we proposed to use AUC > 0.80 as a criterion for defining systems biomarker candidates. Hepatic encephalopathy and lung fibrosis had the AUCs (0.89, 0.80), sensitivities (0.73, 0.56), and specificities (0.90, 0.91) among 48 human diseases (Fig. 3).

Evidence suggested that metabolically impaired brains, including hepatic encephalopathy, biologically compensated for increased lactic acid metabolism^27,28^. Hyperammonemia is important pathogenesis of hepatic encephalopathy. Moderate grade hyperammonemia activates lactate dehydrogenase-4 and 6-phosphofructo-2-kinase to support increased lactate turnover in the brain slices^29^, which was consistent with our finding.

The serum LDH activities were also a decent systems biomarker candidate for lung fibrosis, as shown in Fig. 3. Published reports suggested that the elevated serum LDH in interstitial lung disease could be a predictive factor for the onset of acute exacerbation in scleroderma lung. The elevation of LDH might indicate lung fibrosis^30,31^. Remarkably, a study showed that high serum LDH is a positive predictor of adverse outcomes in critical COVID-19 patients. The highest LDH value in the fibrosis phase of non-survivors is higher than those in survivors^32^. These observations suggested that elevated serum LDH in lung fibrosis patients might be associated with the inflammatory response.

LDH activities are peaked at 3–4 days and remain elevated for up to 10 days following a myocardial infarction, indicating extra serum LDH is removed slowly in the blood circulation. However, the molecular mechanism responsible for the clearance of serum LDH is largely unknown. Remarkably, the data in Fig. 2 showed that the blood-related cancers and diseases, including myeloproliferative disorders, aplastic anemia, anemia, leukemia, and multiple myeloma, were accompanied with significantly higher and lower LDH activities compared to the healthy control and other diseases. These results suggested that these diseases overactivated a molecular mechanism of serum LDH removal at some point of the disease progression. Moreover, the LDH activity distribution in the different classes of diseases had different clustering patterns based on the two major component analyses (Fig. 2), indicating the magnitude of changes in serum LDH levels was differently regulated in various classes of diseases. Thus, understanding the meaning of the clustering phenomena would provide a new direction in understanding systems diseases and systems biomarkers in the near future.

## Methods

### Quantification assays for serum LDH

The method to analyze serum LDH activities is a spectrophotometry-based analysis^33^. LDH catalyzes a reversible conversion of lactate to pyruvate with the conversion of NAD + to NADH. The reaction favors conversion of pyruvate to lactate when the pH is between 6.0 and 7.5 while the reverse applies under the condition that pH is greater than 7.5. NAD+ and NADH have the maximum absorption peaks at 260 nm and 340 nm, respectively. The LDH activity level is measured spectrophotometrically based on the absorbance change of the NADH at 340 nm. Under normal circumstances, LDH activity level in serum is 1000 times lower than in cells or tissues. Therefore, blood samples used for LDH quantification should avoid hemolysis. Two methods perform photometric determinations of LDH. One is monitoring the NAD+ absorbance reduction at 340 nm. The other is a kinetic determination for LDH activity level based on the oxidation rate of NADH^34^. In addition, based on fluorescent NADH to non-fluorescent NAD+, fluorescent capillary analysis technology (FCA) is used to determine LDH activity level^34^.

The clinical lab in our hospital used a LDH assay kit (Lactic acid substrate method, Beijing Leadman Biochemistry Joint stock limited company, Beijing, China) for serum LDH activity measurement according to manufacturer's instructions, which is performed by monitoring the NAD+ absorbance reduction at 340 nm.

### Participants

After obtaining approval from the Hospital Ethics Review Board of Qingdao University, we were allowed to retrieve the electronic medical records and lab data of serum LDH activities of both healthy individuals and patients with clinically defined diseases from the clinical laboratory of the Affiliated Hospital of Qingdao University during the past 5 years. All research was performed following relevant guidelines/regulations, and informed consent was obtained from all participants and/or their legal guardians. The current study retrieved 172,933 clinical lab results of LDH from 48 different types of diseases and 9528 independent tests from individuals during their annual physical examination as the healthy control. Each type of disease had more than 90 independent testing results for serum LDH activities.

### ROC analysis

ROC curves were plotted using SPSS v26 (IBM, Armonk, US). Youden's indices were calculated using ROC curve coordinates to determine AUCs, accuracy, sensitivity, and specificity at the point where test performance is optimal.

### Statistical analysis

The statistical analysis method was similar to that in our previous publication^35^. In brief, all data were analyzed with RStudio V.1.3.1073 (RStudio, Boston, USA), python libraries 3.8 (Anaconda Software Distribution). Values were presented both as median and means ± standard deviation (SD). Standard t-test was used to compare the clinical characteristics of subjects in the specific disease and control groups. Median levels of serum LDH activities between groups were compared by means of the Mann–Whitney U-test. Groups were compared using the Kruskal–Wallis test (a non-parametric one-way ANOVA). Logistic regression was used to test the interactive effects of other variables on the observed association. *p* < 0.05 was considered to be statistically significant.

## Supplementary Information

Supplementary Information.

## Data Availability

All data generated or analyzed during this study are included in this article and/or associated with supplementary information files. Raw data files are available upon request. Correspondence and requests for raw data files should be addressed to L.T. and L.Z. *Data analysis*. Correspondence and requests for detailed data analysis should be addressed to Y.G. and L.Z.
