# Supplementary material for: Serum lactate dehydrogenase activities as systems biomarkers for 48 types of human diseases
Source: Sci Rep. 2021 Jun 21;11:12997. doi: 10.1038/s41598-021-92430-6 (PMC8217520; doi:10.1038/s41598-021-92430-6)
Supplement: Supplementary file 1 — Supplementary Information. [file 41598_2021_92430_MOESM1_ESM.pdf]

Abnormal Serum Lactate Dehydrogenase Activities as Systems Biomarkers for 48 Types of Human Diseases

Yuling Wu[a,b], Caixia Lu[b], Nana Pan[b], Meng Zhang[a], Yi An[b], Mengyuan Xu[b], Lijuan Zhang[a]*, Yachong Guo[c, d]*, and Lijuan Tan[b]*

[a]Systems Biology & Medicine Center for Complex Diseases, Center for Clinical Research, Affiliated Hospital of Qingdao University, Qingdao, 266003, China;

[b]Department of Cardiology, Affiliated Hospital of Qingdao University, Qingdao, 266003, China

[c]Kuang Yaming Honors School, Nanjing University, Nanjing 210023, China

[d]Institute Theory of Polymers, Leibniz-Institut für Polymerforschung Dresden, Dresden, 01069, Germany

***Corresponding authors:**

Lijuan Tan, Yachong Guo, and Lijuan Zhang

Department of Cardiology, The Affiliated Hospital of Qingdao University, Qingdao, 266003, China

Systems Biology & Medicine Center for Complex Diseases, Center for Clinical Research, Affiliated Hospital of Qingdao University, Qingdao, 266003, China

Tel. +86 0532-82031615

Emails: qdtanlijuan@126.com, yguo@nju.edu.cn, zhanglj@qduhospital.cn

Supplemental Figure

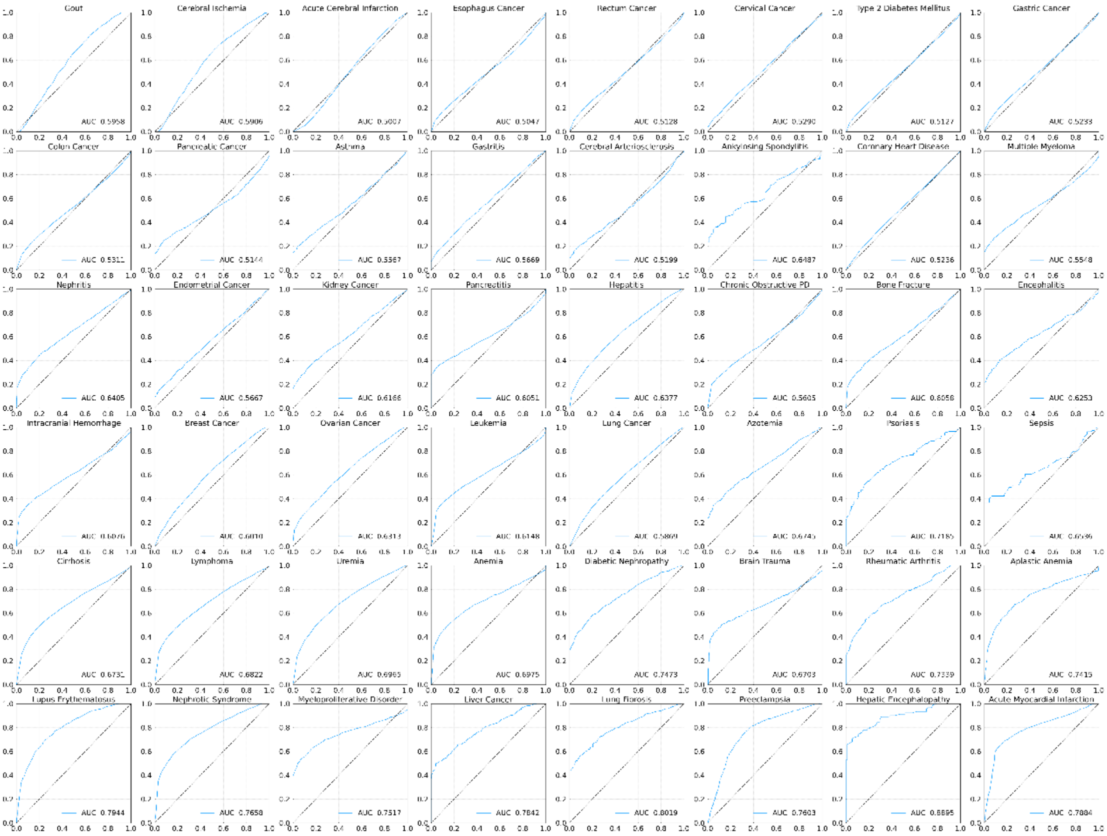

Figure 1. The receiving operator curve (ROC) analysis for all 48 types of diseases. Chronic Obstructive PD: Chronic Obstructive Pulmonary Disease, AUC : Area under the curve.
